# Intelligent Data Management and Security in Cloud Computing

**DOI:** 10.3390/s20123458

**Published:** 2020-06-18

**Authors:** Lidia Ogiela, Marek R. Ogiela, Hoon Ko

**Affiliations:** 1 Department of Cryptography and Cognitive Informatics, Pedagogical University of Krakow, 30-084 Kraków, Poland; lidia.ogiela@up.krakow.pl; 2 Cryptography and Cognitive Informatics Research Group, AGH University of Science and Technology, 30-059 Kraków, Poland; 3 IT Research Institute, Chosun University, Gwangju 61452, Korea; skoh21@chosun.ac.kr

**Keywords:** cryptographic sharing algorithms, data security, service management processes

## Abstract

This paper will present the authors’ own techniques of secret data management and protection, with particular attention paid to techniques securing data services. Among the solutions discussed, there will be information-sharing protocols dedicated to the tasks of secret (confidential) data sharing. Such solutions will be presented in an algorithmic form, aimed at solving the tasks of protecting and securing data against unauthorized acquisition. Data-sharing protocols will execute the tasks of securing a special type of information, i.e., data services. The area of data protection will be defined for various levels, within which will be executed the tasks of data management and protection. The authors’ solution concerning securing data with the use of cryptographic threshold techniques used to split the secret among a specified group of secret trustees, simultaneously enhanced by the application of linguistic methods of description of the shared secret, forms a new class of protocols, i.e., intelligent linguistic threshold schemes. The solutions presented in this paper referring to the service management and securing will be dedicated to various levels of data management. These levels could be differentiated both in the structure of a given entity and in its environment. There is a special example thereof, i.e., the cloud management processes. These will also be subject to the assessment of feasibility of application of the discussed protocols in these areas. Presented solutions will be based on the application of an innovative approach, in which we can use a special formal graph for the creation of a secret representation, which can then be divided and transmitted over a distributed network.

## 1. Introduction

The processes of intelligent data management and securing are currently focused on combining the processes of safe storage of secret, confidential or strategic and developmental information with the processes of secret data management. The data protection process is not the execution of a secret information complete securing process, but subject also to the processes of storage or distribution [1,2,3,4,5,6].

Currently, the most important role in the data-management process is played by the stage of data protection (securing) [7,8,9,10]. Data with great importance-strategic or secret-are subject to special protection. Joint implementation of the security processes relating to the data protection in entities responsible for their protection is always subject, to a greater or smaller extent, to attempts of attacks on significantly important data [11,12]. An attempt to handle this kind of risk is to use cryptographic data-sharing techniques. Among the cryptographic data sharing techniques we can differentiate a class of information sharing protocols, within which cryptographic schemes are applied implementing the processes of splitting data among a group of secret trustees (from among whom a specified number of protocol participants can re-produce the shared secret). The type of the applied protocol determines the way in which a secret is split by means of a specified algorithm, similar to the way re-creating the secret is also determined by an appropriate algorithm. Among the most frequently used cryptographic protocols executing the tasks of secret sharing are the following algorithms [13,14,15,16,17]:

- Tang’s algorithm,
- Lagrange’s interpolation polynomial algorithm,
- Shamir’s algorithm,
- Asmuth–Bloom algorithm.

Secret-sharing protocols guarantee data protection by means of dividing it and splitting secret parts among the protocol participants. The number of protocol participants is defined at the stage of selection of the data-sharing algorithm. It is important in this respect whether the co-shared secret parts will be allocated with equal rights to its trustees, or whether the splitting will privilege some. The Shamir algorithm is an example of a protocol with equal shadow division [13,15], while the Tang algorithm is an example of a protocol with privileged shadows [17]. In the Tang algorithm, there is a specification of such a secret trustee group who, while having the number of shadows smaller than the protocol requires, can re-create the original message. 

In references [2,18], new methods of shadow allocation have been proposed with such protocols, in which it is possible to specify the privileged shadows. It has been specified that not only is it possible to re-produce the information by the privileged protocol participants, but that the very process of allocation of the shared parts can be, to a certain extent, privileged. A specified group of protocol participants can obtain a number of shadows higher than the remaining participants. Such a solution can occur in a situation in which the secret is shared in hierarchical structures, when protocol participants have varied (different) access rights to the secret information or when they have different knowledge about the concealed data. All possibilities of splitting the shared secret have been described in, among others, the following works [2,18].

Also new management solutions were proposed [19,20] oriented for cloud-edge environment and internet of things (IoT) technologies. Further presented approach will also be oriented for secure data transmissions and management is such environments. 

The objective of this paper is presentation a new approach for application of linguistic methods for secret-sharing protocols and data management in hierarchical structures. The main goal is to describe how different linguistic and graph formalisms can be applied for secure information division and management. Application of linguistic formalisms allows the creation of additional secret parts connected with formal grammars used for secret division. Such a solution is a novel approach which allow to create very universal division protocols, which operate on several levels in hierarchical structures. On each level we can consider a different number of trusted persons and different threshold parameters used for secret sharing. Such universality is the main advantage of the proposed solution in relation to traditional data division techniques.

The novelty described in this paper is the whole protocol of intelligent secret management and securing it with the use of data sharing techniques, as well as linguistic description of the concealed secret meaning.

This protocol allows simultaneous data splitting and management to be performed in hierarchical structures. Traditional splitting techniques usually generate secret parts with the same importance and privileges. In the proposed solutions, it is possible to generate secret parts with higher importance than others and also different accessing grants can be generated for users located at different levels in a management structure. Such a protocol is very universal and computationally efficient, and can be applied for different management tasks. As a scientific solution it is not dependent on local security requirements, and can be applied in a broad range of secure information management applications.

The structure of this paper is the following. In Section 2 we shall present our original method of linguistic data description. In Section 3 we shall discuss, proposed by the authors of this paper, intelligent secret management and protection algorithms. In Section 4 we shall present the possibilities of application of the solutions discussed here. In the last section of this paper, we shall summarize the findings and present the directions for further development of the data protection procedures discussed here.

## 2. Linguistic Secret Description

The notation of a data subject to the concealment processes can take various forms; the form of the notation is meaningful for the concealment process. Confidential data noted in the encrypted form become illegible for an ordinary user, but they can be decrypted if the encoding protocol can be broken. That is why the very process of data notification in the encrypted form does not guarantee full security. The data concealment process presented in this paper is subject to the application of cryptographic protocols for secret sharing [2,18].

A novelty proposed by the authors in this paper is the possibility to introduce linguistic description of the concealed data, aimed at extracting the important data and its meaning from the entire set of information concealed. The proposed process of linguistic description depends on the number of secrets shared, in the set of which it is possible to specify the following divisions:

- dividing a single secret piece of information,
- dividing a secret set.

In the first case, the algorithm for how to proceed is as follows (Algorithm 1):
**Algorithm 1:** Dividing a single secretStep 1. Obtaining the secret information. Step 2. Specifying the important features of the concealed secret:—linguistic description of the concealed information meaning,—impact assessment of the concealed secret on the internal and external situation of the entity who has the secret.
Step 3. Selection of secret-sharing protocol:—specifying the type of secret sharing–equal/privileged allocation —specifying the required number of protocol participants, dependent on the type of secret sharing applied.
Step 4. Execution of the secret-sharing protocol:—allocation of shadows to protocol participants,—putting together the required number of shadows to re-produce the secret. 

In the second case, the following algorithm is implemented (Algorithm 2):
**Algorithm 2:** Division of the secret setStep 1. Obtaining the information set. Step 2. Assessment, which information from the obtained set are strategic/secret and are subject to the concealment procedure:—obtaining the secret data from the entire data set,—removing insignificant data or using them to ‘distort’ the meaning of the important information.
Step 3. Obtaining the secret data set. Step 4. Specifying the important features of the concealed data:—in the case of specifying secret data with homogeneous meaning, it is possible to introduce a similar/joint linguistic description for such data,—in the case of data with various meanings, the introduction of independent linguistic descriptions for individual secrets,—impact assessment of the concealed secret on the internal and external situation of the entity who has the secret.
Step 5. Selection of the secret-sharing protocol:—specifying the type of secret sharing-equal/privileged allocation,—specifying the required number of protocol participants.
Step 6. Execution of the secret-sharing protocol:—allocation of shadows to protocol participants,—putting together the required number of shadows to re-produce the secret.

The algorithms presented are to provide linguistic description of the concealed secret data. At the same time, the linguistic description guarantees the assessment of the importance degree (meaningfulness) of the concealed secret. Based on a complete analysis of the secret data, it is possible to determine the meaning of the concealed secret in reference to a given entity, globally, as well as to evaluate the forecasts and determinants shaping the situation assessed and the form of the secret data. 

The linguistic description of a given phenomenon or a data set (in particular secret data) can be executed based on the application of linguistic techniques. Classical linguistic techniques are based on the application of grammar formalisms, in particular of sequential, tree or graph grammars [21,22]. 

The solutions based on the application of sequential grammars are based on a simple secret description in the form of a sequence. Solutions more complex take the form of a tree or a graph [22]. These solutions were developed based on the application of various class formal grammars proposed by Noam Chomsky, according to the following classification [23]:

- unrestricted grammars (type 0);
- context grammars (type 1);
- context-free grammars (type 2);
- regular grammars (type 3).

**Figure 1:**
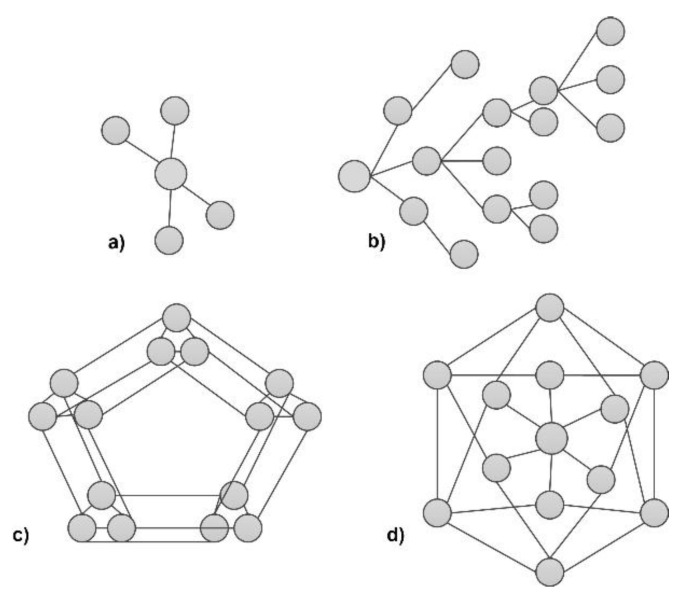
Examples of tree (**a**,**b**) and graph (**c**,**d**) structures applicable in data concealment processes.

Selected tree and graph structures applied in the data concealment processes by means of splitting them are presented on Figure 1. When we consider graph as a structure for data division, we can consider in most cases simple graphs with node labeled, and undirected edges. It is possible of course applied more complex hierarchical graphs like IE (Indexed Edge) graphs [24], but for proper secret representation it is not necessary especially introduced edge-directed structures.

Examples of selected structures presented on Figure 1 show how it is possible to make connections between individual structures’ elements. The way of combing individual elements of a given structure determines the form of possible connections between the participants of data-sharing protocols. A grammar structure becomes the starting point for the entire definition process for the secret-sharing protocol. 

Re-creation of individual elements of selected structures can be moved to the area of secret concealment process, while the very method of possible connections between individual hubs can point to the method of secret part division and allocation to protocol participants. Moreover, it can determine the method in which the original information is re-produced. 

## 3. Intelligent Algorithms of Data Management and Security

This section will discuss the novel intelligent data security algorithms proposed by the authors, and dedicated to data management processes. All methods described in this paper have been proposed by the authors to enrich the existing solutions in the field of data protection.

Intelligent data management and protection algorithms serve the purposes of executing the processes of protecting data against unauthorized access. Data protection protocols have been drafted in order to protect various types of secret information with secret/strategic features. A special type of data described in this paper are services, treated also as a type of data subject to protection and securing. Every piece of information referring to the processes of service management processes with strategic character (developmental) is subject to the process of information protection and concealment; these processes are executed pursuant to the application of threshold schemes extended by the stage of linguistic description of the services secured. For this purpose a security protocol is executed; its implementation depends on the selection of the linguistic type of the analyzed services. There are three types of linguistic threshold schemes:

- sequential threshold schemes;
- tree threshold schemes;
- graph threshold schemes.

Linguistic threshold schemes work pursuant to the following algorithm (Algorithm 3):
**Algorithm 3:** Linguistic threshold schemes.Step 1. Defining (selecting) the service (or information specifying a given service) subject to the concealment process. Step 2. Specifying important characteristic features of the concealed service:—linguistic description of a concealed set by means of a sequential/tree/graph formalism,
Step 3. Execution of the services sharing protocol:—specifying the type of division of the concealed services-equal/privileged allocation —selecting the (*m*, *n*)-threshold scheme,—specifying the *n* of all the protocol participants,—defining the *m* minimal number of shadows required to re-create the secret
Step 4. Execution of the services sharing protocol:—allocation shadows among all the protocol participants,—specifying the minimum number of shadows necessary to re-produce the shared information,—reproducing the concealed information by means of putting together the required number of shadows.

The most important stage of the service concealment process is the stage of its linguistic description. At this stage, the most important features of the analyzed set are defined. A set of all possible features which can be specified, characteristic for the analyzed data sets, is unambiguously defined. As a result of the description applied, every service in the understanding of confidential data (or set of services) is described in an unequivocal manner, with no possibility of ascribing a given feature/features to other service sets. The clarity of the description facilitates an appropriate identification of the set of services and, at the same time, every set of characteristic features is, in an unambiguous way, ascribed to a given set of services. The extraction of characteristic features of the concealed data set makes it possible to describe the meaning (importance) of the analyzed secret data sets. The method of secret information description depends on the selection of a linguistic description. The method of selecting the linguistic description in new classes of cryptographic protocols determines also the method of execution of the secret-sharing protocol. 

## 4. Possible Application of Proposed Linguistic Threshold Schemes

The application of the discussed procedures of data-services concealment according to the Authors of proposed techniques, can be varied. The most important applications of the discussed linguistic threshold procedures are:

- protecting and safeguarding data-services against unauthorized acquisition,
- managing the secret and individual parts of the shared secret,
- distribution of the concealed data among protocol participants and among individual structures of the data management-the level of organization (a given organizational structure), the superior level of ‘fog’ and the cloud level,
- protection of strategic data of any content-the applied secret securing protocols can be used independently of the content and meaning of the concealed information,
- a possibility to apply intelligent threshold schemes for the description of the concealed data meaning,
- a possibility to apply threshold techniques for the tasks of secret management in various organizational structures-hierarchical, layer and mixed.

**Figure 2:**
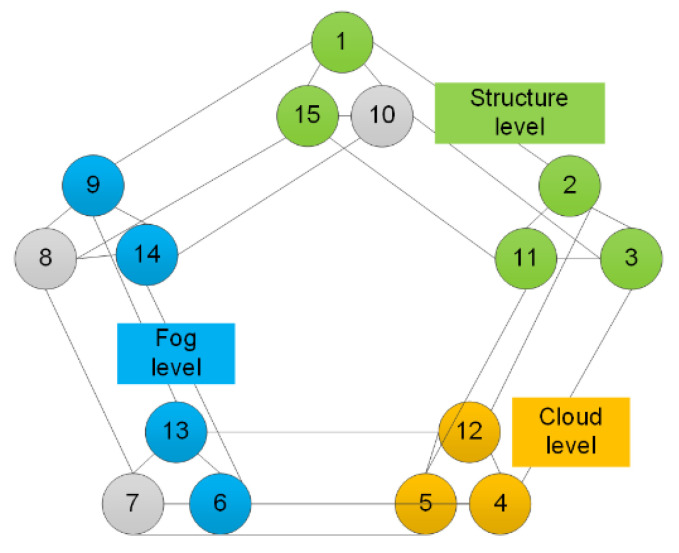
The scheme of secret data management in a hierarchical structure.

Intelligent data-services management procedures-due to the possibility that they can be used in hierarchical structures, are applicable in the data protection and securing processes in cloud computing. A special type of application of the discussed protocols to the processes of data management in a cloud results from a possibility to execute the procedures of concealing data in hierarchical structures. Figure 2 presents a schematic presentation of the discussed application of intelligent linguistic threshold schemes in a hierarchical structure.

The method presented on Figure 2, i.e., a definition of a linguistic graph offers a possibility to define the threshold scheme for a hierarchical structure. An example of such a structure is a three-level secret management process, where at the lowest level there is the level of an organization-the body that possesses the concealed secret; one level higher is the fog level from which it is possible to manage the shared secret both taking into consideration the division of data at a higher and lower level. The third level is the cloud level; it is superior to the two previous ones. At every level of managing the shared secret, an independent scheme of secret sharing is executed. At the lowest level, the group of protocol participants necessary to reproduce the original message is the largest—Figure 2 presents it as a (4, 5)-threshold scheme; at the fog level that number is the smallest—Figure 2 shows it as a (3, 4)-threshold scheme; at the cloud level it is possible to re-create the secret by the least numerous group of shadow holders, i.e., in a (2, 3)-threshold scheme. Such a way of privileged secret re-production results from the use of various shadow distribution schemes at various secret management levels.

**Figure 3:**
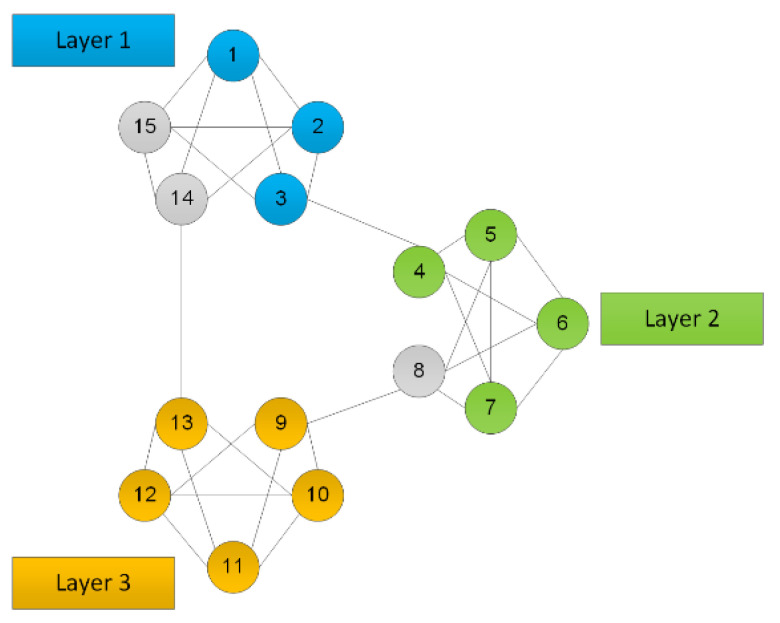
The scheme of secret data management in a layer structure.

Another example of application of the graph structure in the process of linguistic definition of threshold schemes is the layer structure. An example of application of such a scheme in the procedure of secret concealment and management is shown on Figure 3.

The secret-sharing scheme in the layer structure, presented in Figure 3, shows three layers within which there are independent secret sharing procedures executed. In every layer there is a possibility to have an independent description of concealed data taking into consideration various sets of features characteristic for these sets. It is also possible, within every layer, to apply various threshold schemes to reproduce the secret. The graph structure suggest various possibilities of both description and distribution of the shared secret shadows. In every layer a different threshold scheme is executed. In the first layer it is the (3, 5)-threshold scheme, in the second one a (4, 5)-threshold one, while in the third layer it is a (5, 5)-threshold scheme. Presented in Figure 3 sub-graphs define the possibilities to restore the same secret information using different threshold values (designated with different node colors). Depending on the layer configuration different numbers of participants have abilities to restore the original secret data. At each layer different numbers of secret parts are necessary for final data reconstruction.

The layer of linguistic description and division of a secret can be characteristic for the secret management processes at various levels-at the level of a given structure, at the fog level and at the cloud level. In the example presented here, layers do not penetrate each other and the processes executed are dedicated to each layer independently.

The presented specification of secret data management process points to various possibilities of application of the data-sharing protocols discussed here. The most important of them are:

- a possibility to apply independent linguistic descriptions of the concealed secrets at various levels of the management processes-independent methods of linguistic description can occur at the level of the structure, fog and cloud;
- application of independent data-splitting processes within individual management processes-at every level it is possible to apply an independent secret division;
- a possibility to distribute shadows of the shared secret among a group of secret trustees from a given structure level, or to the protocol participants who are at various levels (i.e., at the level of structure, fog or cloud).

The innovative applications of linguistic threshold schemes defined in this paper concern the possibility of using them with the use of various types of formal grammars as well as at various levels of data-service management.

**Figure 4:**
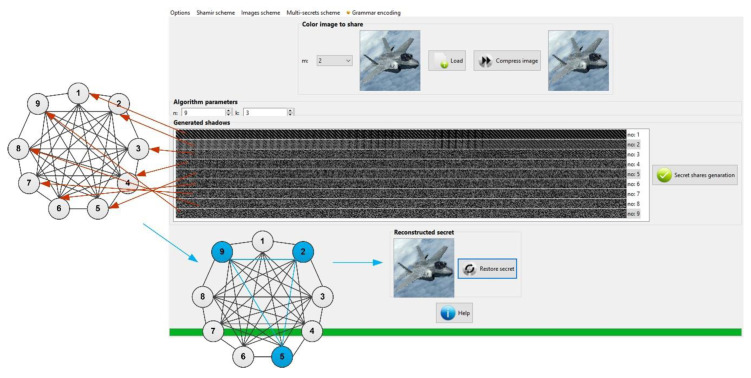
An example of application of linguistic threshold scheme with using the graph structure.

An example of the use of the discussed solutions is shown in Figure 4.

The example in Figure 4 shows how to divide a secret message using a visual (3, 9)-linguistic threshold scheme. The secret message in the form of the image has been subjected to a process of encryption using a linguistic graph-based threshold scheme, and divided into nine parts. Each of the nine nodes of the graph illustrates the secret part generated in the process of dividing the information. They are, therefore, clearly assigned to particular parts of the secret. 

To reproduce a shared secret presented in the source image, it is necessary to collect any three secret parts from nine available. In the example from Figure 3, these are the shadows numbered 2, 5, 9.

The shadow numbers clearly indicate the numbers of the graph nodes, on which the full and closed graph opens, giving the possibility to read the secret information. Visible secret parts have the forms of gray bars generated using threshold procedure dedicated for visual pattern sharing. During collection of such visual shares we can reveal the original image only after providing the required number of parts, necessary to restore the original information. 

Graph threshold schemes can be used in both hierarchical and layered structures. The type of structure determines the form of a graph, that can be used in secure and secret management processes.

Linguistic threshold schemes due to polynomial computational complexity are optimal methods for securing confidential data.

## 5. Results

For testing the presented linguistic approaches two different information management structures were considered. The first one was layered structure having 10 different layers. At each layer were defined between 6 to 10 instances (participants) for which example data were divided and distributed. In the case of a layered structure, each level was independent so in such cases was only possible to test secret recovery using graph approaches defining different threshold values (m, n) connected with particular level. Even for division of the secret data in a different manner for different layers, presented in this contribution, the approach allows original information to be properly restored. For each layer were considered different threshold parameters like presented on example described in Figure 3.

A second approach, considered stricter, was a hierarchical tree having also 10 layers with different numbers of nodes at each levels ranging from 6–10. This structure was similar to the layered structure with additional connections between particular layers (as in Figure 1b). The performed tests were connected with several secret data divisions and distribution of obtained secret parts over this hierarchical network in such manner each level allow secret data (with different threshold parameters) to be independently restored, but additionally some secret parts can be replaced by shadows originating from other levels. This situation is presented on the example from Figure 4.

Finally, for the defined layered and hierarchical structure different graph models were introduced and tested, considering different numbers of participants at particular levels. During the conducted tests for secret data division, the system presented in Figure 4 was applied, and 20 different images originating from open image databases were considered.

The goal of the conducted research was comparison of linguistic procedures in layered and hierarchical structures. In the test conducted we tried to prove the universality of proposed techniques, and possibilities of its application in both types of management structures which can be implemented in cloud-distributed systems. The principal objectives were to use different linguistic formalisms i.e., sequential grammar and tree grammars for secret division, and comparison of complexity of such solutions. The test objective was also to check the advantages of application of graph approach in splitting secret data in various manners for different layers in hierarchical structures. Such objectives were confirmed and proved on provided test data. The only one limitation for graph structure representation is higher complexity of secret restoration compared to sequential solutions.

The experiment performed should confirm the possibilities of the application of linguistic formalisms in secret-sharing procedures. Conducted research proved such possibilities, and broad range of new classes of sharing protocols. We successfully implemented an application which allow linguistic modules to be defined for data division, and which also generate secret parts for divided information. All security features are guaranteed by native threshold algorithms used in linguistic protocols. Validation of proposed methods were done on test data sets.

## 6. Discussion

Intelligent data management and protection processes in cloud computing focus on the execution of cryptographic data protection protocols. In this group of protocols an important role is played by the secret-sharing protocols enriched by the process of linguistic description of the concealed data meaning. The processes of linguistic meaning description of the protected strategic information can be executed with consideration of various levels of semantic knowledge relating to the concealed secrets; at the same time, the method of meaning description implies the selection of an appropriate grammar structure. It is possible to have a simple grammar in the form of sequential structure, but also a more complex one in the form of a tree or graph structure. The graph structure shows the greatest possibilities of application in, among others, the area of cloud computing. Due to the character of a graph, in the graph structures it is possible to execute an independent linguistic description at various management levels both in layer and hierarchical structures. Every structure presented here is an example of a three-level secret management process, typical for the levels of organization, fog and cloud. The universality of the solutions presented here allows them to be applied in various areas of management and protection of secret/confidential data. It can also be performed on parallel architecture, which allows secret data for different layers having separate graph representation to be independently split. 

The presented approaches can also be connected with deep-learning technologies used for complex pattern classification. Deep learning allows feature vectors connected with semantic description of analyzed data to be evaluated. Such parameters can be used to determine the optimal configuration for the creation of graph representation. Based on such parameters, data can be shared over distributed networks.

The open issue for the presented methods is automatic generation and formal reasoning of new graph structures for secret representation. This is very important in the creation of special 3D representation-reflected different layers in the management structure, for which a secret should be divided and distributed. 

The techniques of intelligent data management and protection in cloud computing can be further developed in various areas and applied to secure data in the form of CAPTCHA (Completely Automated Public Turing test to tell Computers and Humans Apart). This also can be extended towards the application of distributed matching keys described in [25,26] or steganography [27]. These directions of research show the future for investigative work.
